# Generation of Transgenic Self-Incompatible *Arabidopsis thaliana* Shows a Genus-Specific Preference for Self-Incompatibility Genes

**DOI:** 10.3390/plants8120570

**Published:** 2019-12-04

**Authors:** Tong Zhang, Guilong Zhou, Daphne R. Goring, Xiaomei Liang, Stuart Macgregor, Cheng Dai, Jing Wen, Bin Yi, Jinxiong Shen, Jinxing Tu, Tingdong Fu, Chaozhi Ma

**Affiliations:** 1 National Key Laboratory of Crop Genetic Improvement, National Center of Rapeseed Improvement in Wuhan, Huazhong Agricultural University, Wuhan 430070, China; 2 Department of Cell & Systems Biology, University of Toronto, Toronto, ON M5S 3B2, Canada; 3 Centre for Genome Analysis & Function, University of Toronto, Toronto, ON M5S 3B2, Canada

**Keywords:** self-incompatible, *SCR* (*S-locus Cysteine-Rich*), *SRK* (*S* locus receptor kinase), *ARC1* (Arm repeat containing), *Arabidopsis thaliana*, genus-specific preference

## Abstract

*Brassicaceae* species employ both self-compatibility and self-incompatibility systems to regulate post-pollination events. *Arabidopsis halleri* is strictly self-incompatible, while the closely related *Arabidopsis thaliana* has transitioned to self-compatibility with the loss of functional *S*-locus genes during evolution. The downstream signaling protein, ARC1, is also required for the self-incompatibility response in some *Arabidopsis* and *Brassica* species, and its gene is deleted in the *A. thaliana* genome. In this study, we attempted to reconstitute the SCR-SRK-ARC1 signaling pathway to restore self-incompatibility in *A. thaliana* using genes from *A. halleri* and *B. napus*, respectively. Several of the transgenic *A. thaliana* lines expressing the *A. halleri* *SCR_13_-SRK_13_-ARC1* transgenes displayed self-incompatibility, while all the transgenic *A. thaliana* lines expressing the *B. napus* *SCR_1_-SRK_1_-ARC1* transgenes failed to show any self-pollen rejection. Furthermore, our results showed that the intensity of the self-incompatibility response in transgenic *A. thaliana* plants was not associated with the expression levels of the transgenes. Thus, this suggests that there are differences between the *Arabidopsis* and *Brassica* self-incompatibility signaling pathways, which perhaps points to the existence of other factors downstream of *B. napus* *SRK* that are absent in *Arabidopsis* species.

## 1. Introduction

Self-incompatibility is one of the most important breeding systems present in many hermaphroditic flowering plants as it causes self-pollen rejection to promote out-crossing. Self-incompatibility systems under investigation include species in *Brassicaceae*, *Solanaceae*, *Rosaceae*, *Scrophulariaceae* and *Papaveraceae* [1,2,3,4]. In the *Brassicaceae* (crucifer family), self-incompatibility is controlled by tightly linked multi-allelic genes at the *S*-locus region, which rarely recombine and so have collectively been named *S*-haplotypes [4]. Three *S*-locus-linked genes have been identified in *Brassica* species. The *S*-locus glycoprotein (*SLG*) gene encodes an abundant, secreted glycoprotein that is located in the cell wall of stigma papillae, and SLG has been reported to be connected to pollen adhesion on stigmatic surfaces [5,6]. The *S*-locus receptor kinase (SRK) is the female determinant in stigma, and mediates the self-incompatibility signaling pathway [7,8,9]. Finally, the *S-locus Protein 11/S-locus Cysteine-Rich* (*SP11/SCR*) gene encodes the male determinant and is expressed in the anther tapetum and pollen [10,11,12]. SP11/SCR is recognized by SRK as a ligand in an *S*-haplotype-specific manner, and SRK is then autophosphorylated to induce various signaling cascades [13,14].

Models of the downstream self-incompatibility signaling pathways have been proposed in *Brassica* species [15,16]. The *M* locus protein kinase (MLPK) was identified as a positive regulator of self-incompatible response, since the *B. rapa mlpk* mutant lost its self-incompatibility phenotype and knocking out the *B. napus MLPK* genes by CRISPR/Cas9 completely knocked out the self-incompatibility response [17,18]. MLPK is tethered on the plasma membrane and can be phosphorylated by SRK by forming a complex with SRK to mediate signal transduction in self-incompatibility response [19,20]. ARC1 (Armed Repeat Containing 1) and THL1/2 (thioredoxin-h like 1/2) were isolated for interacting with the kinase domain of SRK in yeast two-hybrid analysis [21,22]. THL1/2 were believed to regulate self-incompatibility response negatively [21,23]. Down-regulation of *ARC1* expression in the stigma caused a breakdown of self-incompatibility in the *B. napus* line ‘W1′ [22,24]. ARC1 is an E3 ubiquitin ligase that can direct the degradation of target proteins required for compatible pollen responses [25]. One target is EXO70A1, which directly interacts with ARC1 and can be ubiquitinated by ARC1 in vitro. This action is proposed to block the transfer of secretory vesicles to the pollen-stigma contact site resulting in pollen rejection [26]. GLO1 (glyoxalase I), a key enzyme involved in detoxification of methylglyoxal, and has been identified as another ARC1 target. ARC1 was proposed to negatively regulate GLO1 in self-pollen rejection, leading to the accumulation of the methylglyoxal toxin in papillar cells [27]. 

In addition to *Brassica* species, *Arabidopsis* species have been used for self-incompatibility studies in *Brassicaceae*. By analyzing the sequences of *Arabidopsis S*-locus genes, naturally self-incompatible species, such as *Arabidopsis lyrata* and *Arabidopsis halleri*, were shown to have intact *SRK* and *SCR/SP11* genes. *Arabidopsis thaliana* ecotypes are self-compatible and do not carry functional *SRK* or *SCR/SP11* genes [28,29,30,31,32]. Efforts have been made to reconstruct the self-incompatibility response in *A. thaliana* by introducing functional *SCR* and *SRK* genes, and significant differences were observed in the stability and strength of the self-incompatibility traits among various ecotypes tested [31,33,34,35,36,37]. When *B. napus* self-incompatibility stigma genes (*SLG*, *SRK* and *ARC1* genes) were transformed into *A. thaliana*, the transgenic plants still produced seeds, and their stigmas failed to reject the corresponding self-incompatible *B. napus* W1 pollen [38]. Similarly, transgenic *A. thaliana* ecotype Col-0 plants expressing the *A. lyrata SCRb-SRKb* genes were fully self-fertile [33,35,39]. However, when the same experiments were conducted with the *A. thaliana* C24 ecotype, the transformation of the *AlSCRb-AlSRKb* genes resulted in transgenic plants showing a strong self-incompatibility response [33,35,40]. Self-incompatible *A. thaliana* C24 lines were also created using the *A. halleri AhSCR_1_* and *AhSRK_1_* genes of the most recessive *S_1_* haplotype [41]. Finally, *ARC1* was found to be a pseudogene in *A. thaliana*, and a homolog of *MLPK*, *AtAPK1b*, did not appear to be involved in regulating self-incompatibility in transgenic *A. thaliana*, suggesting that *SRK-*mediated signaling in transgenic *A. thaliana* might be different from the *Brassica* models [37,42]. This is also supported by differences in the post-pollination cellular responses of *Brassica* and *Arabidopsis* stigmas [15,16]. Nevertheless, stable self-incompatible *A. thaliana* were finally generated in the Col-0 ecotype when *AlARC1* or *BnARC1* were expressed along with the *AlSCRb-AlSRKb* transgenes [43].

Here, we further examine the question of whether *Brassica* and *Arabidopsis* self-incompatibility genes can share a common self-incompatibility signaling pathway in transgenic *A. thaliana* using a new set of self-incompatibility genes from *B. napus* and *A. halleri*. The *B. napus S_1_*-haplotype *SCR/SP11* and *SRK* genes [44,45,46] along with *BnARC1* were transformed into two *A. thaliana* accessions, Col-0 and C24. As both the pollen (*BnSCR/SP11_1_*) and stigma (*BnSRK_1_, BnARC1*) factors were included for this study, this differs from a previous study [38] where only stigma *B. napus* factors were transformed. A new set of *Arabidopsis* self-incompatibility genes from *A. halleri S_13_*-haplotype *SCR/SP11* and *SRK* genes [47], along with *AhARC1,* were also tested into Col-0 as a comparison. The *S_1_-* and *S_13_*-haplotypes were previously found to be the most dominant *S*-haplotypes in *B. napus* and *A. halleri*, respectively [41,44,45]. Our results show that self-incompatibility genes from *B. napus* were not sufficient to restore the self-incompatibility in both the Col-0 and C24 ecotypes, while self-incompatibility was successfully transmitted into Col-0 with the *A. halleri* self-incompatibility genes.

## 2. Results

### 2.1. Reconstruction of ARC1-Mediated Self-Incompatibility in A. thaliana Col-0 using A. halleri Self-Incompatibility Genes

To examine if the *SCR-SRK-ARC1* model is conserved using genes from another naturally self-incompatible *Arabidopsis* species, *A. halleri SCR_13_-SRK_13_* genes were introduced into *A. thaliana* Col-0 plants in the presence or absence of *AhARC1*, with all three transgenes in a single construct. Thirty and 34 independent T_1_ Col-0 lines were generated with the addition of *AhSCR_13_-AhSRK_13_* and *AhSCR_13_-AhSRK_13_-AhARC1*, respectively (Table 1). The self-incompatibility phenotype of the transgenic plants was examined by staining their stigmas with aniline blue to visualize the pollen grains and the pollen tubes growing into papillar cells. Pollinated pistils from the different transgenic lines showed variations in the levels of rejection/acceptance of self-pollen, which was also reported by Indriolo et al. [43]. Accordingly, the phenotypes were defined as self-compatible (similar to Col-0), moderately self-incompatible (>5 pollen tubes/pistil, but visibly reduced compared to Col-0) or strongly self-incompatible (<5 pollen tubes/pistil). 

**Figure 1:**
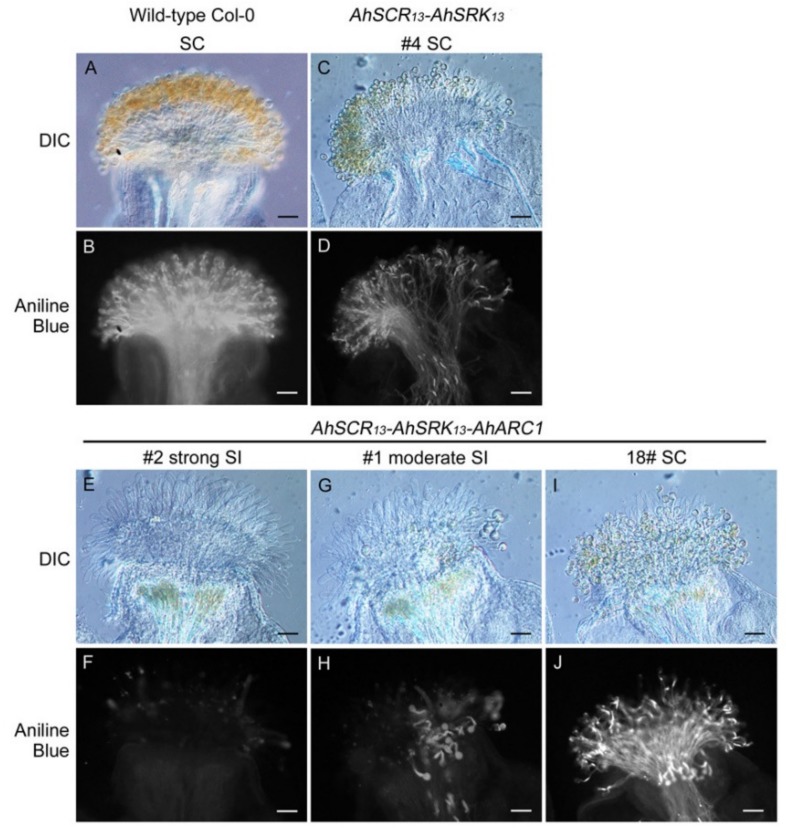
Pollen tube germination and growth in transgenic *A. thaliana* Col-0 plants. (**A,B**) Wild-type *A. thaliana* Col-0 self-pollinated stigma. (**C,D**) Transgenic *A. thaliana* Col-0 *AhSCR_13_-AhSRK_13_* line-4 self-pollinated stigma. (**E–J**) Transgenic *A. thaliana* Col-0 *AhSCR_13_-AhSRK_13_-AhARC1* line-2, -1 and -18 self-pollinated stigmas. Differential interference contrast (DIC) and aniline blue-stained images are shown for each sample. SC: self-compatible; SI: self-incompatible. Bars = 50 μm.

All 30 independent transgenic T_1_ plants with *AhSCR_13_*-*AhSRK_13_* were found to be fully self-compatible, as they could accept self-pollen similarly to that observed for wild-type self-pollinated Col-0 plants in stage-14 stigmas (Table 1 and Figure 1A–D). In contrast, four out of 34 transgenic T_1_ plants carrying *AhARC1* along with *AhSCR_13_-AhSRK_13_* showed strong self-incompatibility (Table 1 and Figure 1E,F). To ensure that the strong self-incompatibility phenotype was not caused by other reproduction factors, reciprocal crosses were performed between the transgenic lines and wild-type Col-0 plants, leading to complete self-compatibility (Supplementary Figure S1). In addition, 16 of the 34 *AhSCR_13_-AhSRK_13_-AhARC1* transgenic T_1_ plants displayed moderate self-incompatibility, and the final 14 *AhSCR_13_-AhSRK_13_-AhARC1* transgenic T_1_ plants were fully self-compatible (Table 1 and Figure 1G–J). The fact that only the combination of *AhSCR_13_-AhSRK_13_-AhARC1* transgenes could reconstruct self-incompatibility in the *A. thaliana* Col-0 ecotype confirms a role for ARC1 in this self-pollen rejection signaling pathway, as previously reported by Indriolo et al. [43].

### 2.2. Self-Incompatibility Related Genes of B. napus are Not Sufficient to Restore Self-Incompatibility in both A. thaliana Col-0 and C24 Ecotypes

**Figure 2:**
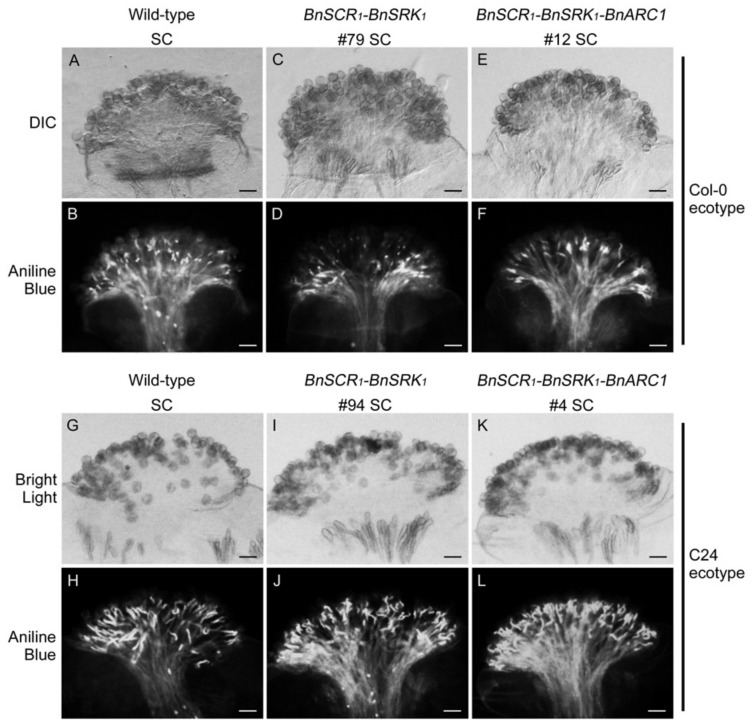
Pollen tube germination and growth in transgenic *A. thaliana* Col-0 and C24 plants. (**A,B**) Wild-type *A. thaliana* Col-0 self-pollinated stigma. (**C,D**) Transgenic *A. thaliana* Col-0 *BnSCR_1_-BnSRK_1_* line-79 self-pollinated stigma. (**E,F**) Transgenic *A. thaliana* Col-0 *BnSCR_1_-BnSRK_1_ -BnARC1* line-12 self-pollinated stigma. (**G,H**) Wild-type *A. thaliana* C24 self-pollinated stigma. (**I,J**) Transgenic *A. thaliana* C24 *BnSCR_1_-BnSRK_1_* line-94 self-pollinated stigma. (**K,L**) Transgenic *A. thaliana* C24 *BnSCR_1_-BnSRK_1_ -BnARC1* line-4 self-pollinated stigma. Differential interference contrast (DIC) (or regular bright light) and aniline blue-stained images are shown for each sample. SC: self-compatible. Bars = 50 μm.

To test whether the *B. napus SCR_1_-SRK_1_* genes were sufficient to restore self-incompatibility in *A. thaliana* plants, and to test if *BnARC1* was also required, the *BnSCR_1_-BnSRK_1_* genes were transformed with or without *BnARC1* into the *A. thaliana* Col-0 and C24 ecotypes. Independent transgenic plants were obtained for each transformation event, and the pollination phenotypes were noted (Table 1 and Table 2). Twenty-one independent T_1_ Col-0 plants were obtained with the transformation of *BnSCR_1_-BnSRK_1_* and all of them exhibited a self-compatible phenotype in stage-14 self-pollinated pistils stained with aniline blue (Table 1 and Figure 2A–D). Similarly, 13 independent T_1_ C24 lines were created with the *BnSCR_1_-BnSRK_1_* transgenes and showed a similar level of self-pollen acceptance to wild-type C24 plants at developmental stage 14 (Table 2 and Figure 2G–J). Previous studies have reported that *A. thaliana* Col-0 plants carrying the *A. lyrata SCRb-SRKb* transgenes were fully self-compatible, while for *A. thaliana* C24 plants transformed with the *SCRb-SRKb* transgenes, self-incompatibility was successfully reinstated [33,35,40]. However, the introduction of *B. napus BnSCR_1_-BnSRK_1_* into C24 plants failed to restore self-incompatibility, indicating that *BnSCR_1_-BnSRK_1_* may need other unknown factors in the self-incompatibility pathway that do not exist in the C24 ecotype.

To examine if the transformation of *BnARC1* along with *BnSCR_1_-BnSRK_1_* could produce a functional self-incompatible pathway in Col-0 and C24 plants, 12 and 9 independent T_1_ Col-0 and C24 plants were generated, respectively. The phenotypes of these transgenic lines were again scored for self-pollen rejection by aniline blue staining. Unexpectedly and in contrast to the *AhSCR_13_-AhSRK_13_-AhARC1* transgenic Col-0 plants, all Col-0 lines carrying *BnSCR_1_-BnSRK_1_-BnARC1* genes showed self-compatible phenotypes (Table 1 and Figure 2E,F). In addition, no self-incompatible or moderately self-incompatible phenotypes were observed in the *BnSCR_1_-BnSRK_1_-BnARC1* transgenic C24 lines (Table 2 and Figure 2K,L). These results indicate that the addition of *BnARC1* with *BnSCR_1_-BnSRK_1_* also does not work to restore the self-incompatibility signaling pathway in the *A. thaliana* Col-0 and C24 ecotypes.

### 2.3. Seed Production is Significantly Reduced in Col-0 Lines Expressing AhSCR_13_-AhSRK_13_-AhARC1

**Figure 3:**
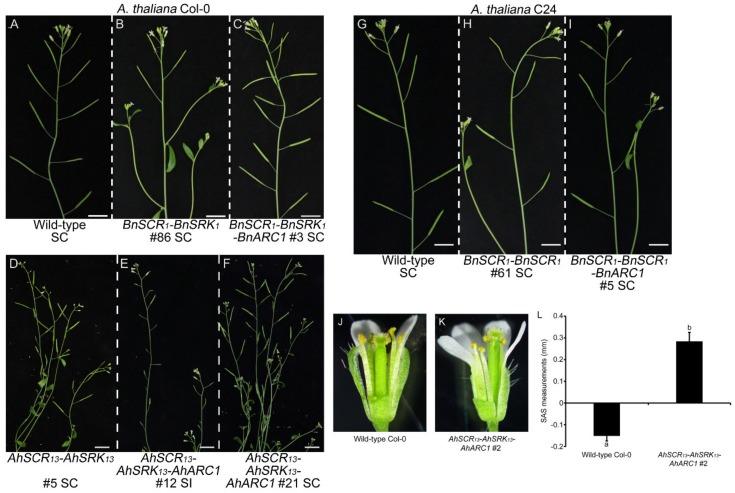
Branches with siliques from transgenic *A. thaliana* Col-0 and C24 plants following self-pollination and the approach herkogamy phenotype in *AhSCR_13_-AhSRK_13_-AhARC1* line 2. (**A,G**) Wild-type Col-0 and C24 branches with well-developed siliques. (**B–F**) Branches with siliques from transgenic *A. thaliana* Col-0 lines: *BnSCR_1_-BnSRK_1_* line 86, *BnSCR_1_-BnSRK_1_-BnARC1* line 3, *AhSCR_13_-AhSRK_13_* line 5, *AhSCR_13_-AhSRK_13_-AhARC1* lines 12 and 21. (**H,I**) Branches with siliques from transgenic *A. thaliana* C24 lines: *BnSCR_1_-BnSRK_1_* line 61 and *BnSCR_1_-BnSRK_1_-BnARC1* line 5. SC: self-compatible; SI: self-incompatible. Bars = 1 cm. (**J**) Wild-type Col-0 and (**K**) A*hSCR_13_-AhSRK_13_-AhARC1* line-2 flowers. Petals and sepals have been removed to allow a clearer view of the anther positions relative to the stigma. (**L**) Stigma-anther separation (SAS) measurements. Col-0 flowers typically have a reverse herkogamy phenotype, which is represented by a negative SAS value. In contrast, the positive SAS value for AhSCR13-AhSRK13-AhARC1 line-2 flowers indicates an approach herkogamy phenotype. SAS measurements were taken as described in Luo and Widmer 2013 [48] and Indriolo et al. 2014 [43]. *n* = 10 flowers.

As the transformation of *SCRb-SRKb-ARC1* was previously found to lead to an approach herkogamous phenotype in *A. thaliana* Col-0 and Sha plants [43], we examined whether the transgenic *SCR-SRK-ARC1* Col-0 and C24 lines displayed any changes in their floral morphology. The approach herkogamous phenotype was only exhibited in the strongly self-incompatible Col-0 plants carrying the *AhSCR_13_-AhSRK_13_-AhARC1* transgenes (Figure 3J,K and Supplementary Figure S2). The physical stigma-anther separation (SAS) was measured in flowers from wild-type and representative self-incompatible Col-0 plants. A positive SAS value was indicative of approach herkogamy which is consistent with the self-incompatible phenotype in the *AhSCR_13_-AhSRK_13_-AhARC1* plants (Figure 3L). However, all the self-compatible transgenic lines showed a normal flower trait with the anthers positioned above the stigmas, similar to wild-type Col-0 and C24 plants, which ensures that the released pollen falls onto the stigmas (Supplementary Figure S2). Therefore, the approach herkogamous phenotype correlates with the degree of self-incompatibility in the transgenic *A. thaliana* plants. 

Since self-incompatibility has been defined as a reproductive mechanism that prevents self-fertilization, seed production of representative transgenic Col-0 and C24 lines was examined. The plants were allowed to self-fertilize naturally, and photographs of branches with siliques from plants with each combination of transgenes are shown in Figure 3. The wild-type *A. thaliana* Col-0 and C24 plants are self-compatible and should produce well-developed siliques full of seeds with self-pollination (Figure 3A,G). The transgenic Col-0 plants with *AhSCR_13_-AhSRK_13_*, *AhSCR_13_-AhSRK_13_-AhARC1*, *BnSCR_1_-BnSRK_1_* or *BnSCR_1_-BnSRK_1_-BnARC1* that did not display self-incompatibility produced siliques with similar sizes to the wild-type Col-0 plants (Figure 3B–D,F). Regular-sized siliques were also observed in the self-compatible transgenic C24 plants carrying the *BnSCR_1_-BnSRK_1_* or *BnSCR_1_-BnSRK_1_-BnARC1* transgenes (Figure 3H,I). However, the strongly self-incompatible transgenic Col-0 plants with the *AhSCR_13_-AhSRK_13_-AhARC1* transgenes displayed much smaller silique sizes, as a result of very reduced seed production with self-pollination (Figure 3E).

**Figure 4:**
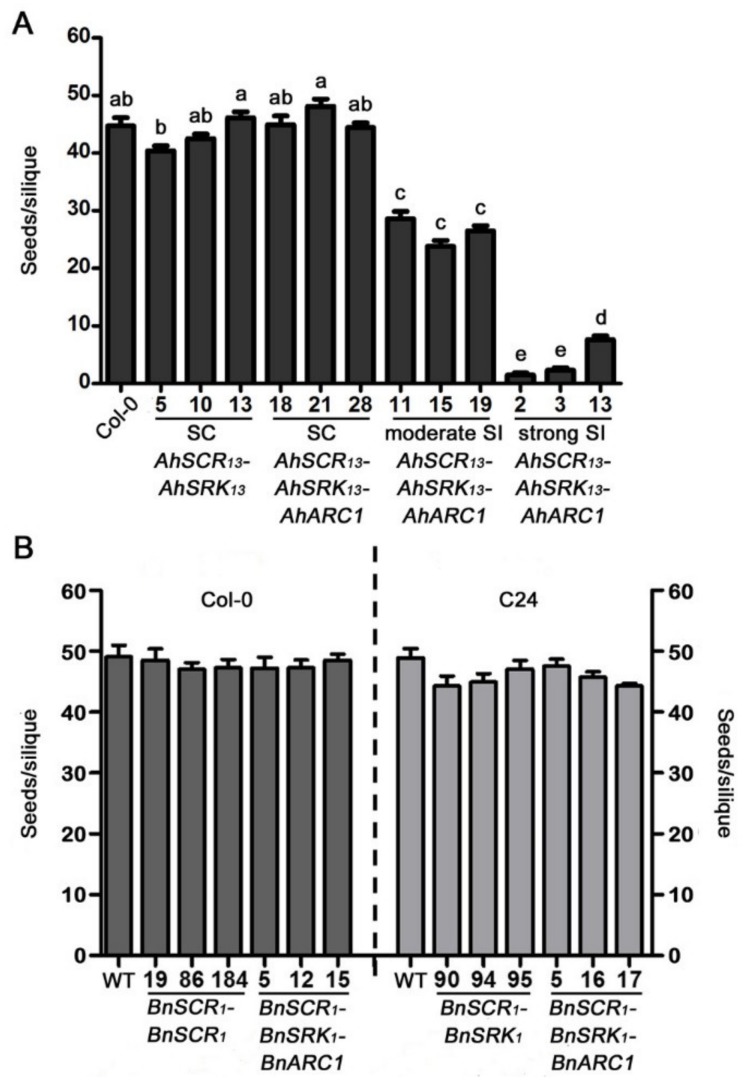
Seed set of transgenic *A. thaliana* Col-0 and C24 plants following self-pollination. (**A**) Mean seeds/silique of self-pollinated wild-type *A. thaliana* Col-0 and transgenic Col-0 *AhSCR_13_-AhSRK_13_* and *AhSCR_13_-AhSRK_13_-AhARC1* lines. Means with significant differences at *p* < 0.05 are shown with different letters (one-way ANOVA with Tukey-HSD post-hoc tests). *n* = 10 siliques. (**B**) Mean seeds/silique of self-pollinated wild-type *A. thaliana* Col-0 and C24 plants, as well as transgenic Col-0 and C24 *BnSCR_1_-BnSRK_1_* and *BnSCR_1_-BnSRK_1_-BnARC1* lines. Significant difference at *p* < 0.05 calculated by one-way ANOVA with Tukey-HSD post-hoc tests. *n* = 10 siliques. Error bars indicate SE. SC: self-compatible; SI: self-incompatible.

Fully developed siliques from each of these lines were harvested and dissected to record the number of seeds per silique (Figure 4). Siliques from wild-type *A. thaliana* Col-0 plants produced an average of 44.7 seeds/silique via self-pollination (Figure 4A). Similarly, the siliques from the three self-compatible *AhSCR_13_-AhSRK_13_* transgenic Col-0 plants contained 40.4, 42.5 and 46.1 seeds/silique on average, respectively, showing no significant difference with wild-type Col-0 (Figure 4A). The siliques of the three self-compatible *BnSCR_1_-BnSRK_1_* and *BnSCR_1_-BnSRK_1_-BnARC1* transgenic Col-0 plants generated similar averages of 48.5, 47, 47.3, and 47.2, 47.3, 48.5 seeds/silique, respectively (Figure 4B). Thus, these self-compatible transgenic Col-0 lines displayed an equivalent ability to accept self-pollen compared to the wild-type Col-0 plants. The addition of *BnARC1* with *BnSCR_1_-BnSRK_1_* did not promote the self-pollen rejection in *A. thaliana* Col-0. In contrast, some reduction in the number of seeds/silique was observed for the three moderately self-incompatible *AhSCR_13_-AhSRK_13_-AhARC1* transgenic Col-0 lines, with averages of 28.6, 23.8, and 26.5 seeds/silique, respectively (Figure 4A). In addition, significant reduction in seed production was observed for the three strong self-incompatible *AhSCR_13_-AhSRK_13_-AhARC1* transgenic Col-0 lines following self-pollination, with the lowest value of 1.5 seeds/silique scored for *AhSCR_13_-AhSRK_13_-AhARC1* line 2 (Figure 4A). Therefore, the expression of *AhARC1* along with *AhSCR_13_-AhSRK_13_* in *A. thaliana* Col-0 induced a strong self-incompatibility response resulting in very reduced seed production.

Mature siliques of natural self-pollination transgenic *A. thaliana* C24 lines were also examined for seed set and compared to wild-type *A. thaliana* C24 plants, which produced an average of 48.9 seeds/silique (Figure 4B). All the self-compatible *BnSCR_1_-BnSRK_1_* and *BnSCR_1_-BnSRK_1_-BnARC1* transgenic C24 lines showed similarly high numbers of seeds/silique following self-pollination, which ranged from 44.3 to 47.5 seeds/silique (Figure 4B). Accordingly, the *B. napus SCR_1_*-*SRK_1_* transgenes in the *A. thaliana* C24 ecotype could not elicit a self-incompatibility response, and the addition of *BnARC1* with *BnSCR_1_-BnSRK_1_* did not result in any reduction of seed set in the C24 ecotype.

### 2.4. The Expression Levels of the SCR-SRK-ARC1 Transgenes are Not Associated with the Intensity of Self-Incompatibility Response in A. thaliana

**Figure 5:**
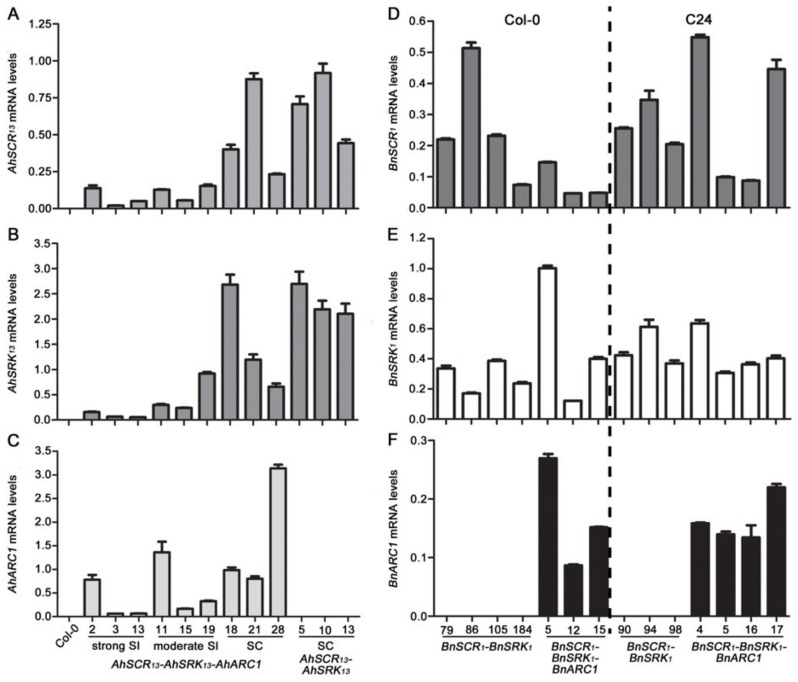
Relative expression levels of *AhSCR_13_*, *AhSRK_13_*, *AhARC1*, *BnSCR_1_*, *BnSRK_1_*, and *BnARC1* in transgenic *A. thaliana* Col-0 and C24 plants. (**A**) Relative mRNA levels of *AhSCR_13_* in mature buds from different lines. (**B**) Relative mRNA levels of *AhSRK13* in mature buds from different lines. (**C**) Relative mRNA levels of *AhARC1* in mature buds from different lines. (**D**) Relative mRNA levels of *BnSCR_1_* in mature buds from different lines. (**E**) Relative mRNA levels of *BnSRK_1_* in mature buds from different lines. (**F**) Relative mRNA levels of *BnARC1* in mature buds from different lines. The relative expression levels of all the genes were normalized to the expression of *Actin2* and *TUB4*. The wild-type Col-0 bud samples were used as a negative control for *AhSCR_13_*, *AhSRK_13_*, and *AhARC1* genes. Primers for qRT-PCR were designed to span introns where possible. Means of two biological replicates (three technical replicates each) are shown. Error bars indicate SE. SC: self-compatible; SI: self-incompatible.

Given the differences observed in the ability of the *AhSCR_13_-AhSRK_13_-AhARC1* and *BnSCR_1_-BnSRK_1_-BnARC1* transgenes to confer self-incompatibility in *A. thaliana*, the transgene expression levels were investigated to reveal if they correlated with the observed phenotypes. Mature buds from representative transgenic lines were harvested to test the relative expression levels of each transgene using quantitative RT-PCR (qRT-PCR). Overall, the transgene expression levels were quite variable, and there was no correlation between a particular transgene expression level and the self-incompatibility trait (Figure 5). For example, the strongly self-incompatible *AhSCR_13_-AhSRK_13_-AhARC1* transgenic Col-0 plants tended to show lower levels of relative expression for all three transgenes (Figure 5A–C). In fact, the highest relative expression level for each transgene was detected in the fully self-compatible *AhSCR_13_-AhSRK_13_-AhARC1* transgenic lines: line 18 (*AhSRK_13_*), line 21 (*AhSCR_13_*) and line 28 (*AhARC1*) (Figure 5A–C). Thus, the expression level of these transgenes did not account for the intensity of self-incompatibility in transgenic *A. thaliana* Col-0 lines.

Although the addition of the *BnSCR_1_-BnSRK_1_* and *BnSCR_1_-BnSRK_1_-BnARC1* transgenes did not restore the self-incompatibility in the *A. thaliana* Col-0 and C24 ecotypes, the expression levels of these transgenes were also quantified to confirm that they were successfully being expressed in the transgenic lines (Figure 5D–F). The *BnSCR_1_* transgene tended to show quite high relative expression levels, particularly in the C24 ecotype (Figure 5D), and the relative levels in both Col-0 and C24 showed similar ranges to that observed for *AhSCR_13_* (Figure 5A). Conversely, the *BnSRK_1_* relative expression levels were generally lower than those observed for several of the transgenic lines carrying the *AhSRK_13_* transgene (Figure 5B,E). However, the *BnSCR_1_-BnSRK_1_-BnARC1* transgenic lines did have *BnSRK_1_* expression levels that were comparable to the *AhSRK_13_* transgene in the strongly self-incompatible *AhSCR_13_-AhSRK_13_-AhARC1* transgenic lines (Figure 5B,E). Finally, the *BnARC1* expression levels were generally higher than the observed relative expression values for the *AhARC1* (Figure 5C,F). Thus, no trend was seen in the various expression levels for the *BnSCR_1_-BnSRK_1_-BnARC1* transgenes that could account for lack of self-pollen rejection in both the *A. thaliana* Col-0 and C24 transgenic plants. This suggests that the *B. napus* self-incompatibility signaling pathway cannot be reconstructed in the *A. thaliana* Col-0 and C24 ecotypes.

## 3. Discussion

The transition to selfing has made the model plant *A. thaliana* an ideal system for the reconstruction of self-incompatibility. It is believed that the loss-of-function mutations in *SRK* and *SCR* genes has led to self-fertility in all *A. thaliana* ecotypes [28,30,31,32,35,49,50,51]. Self-incompatibility has been restored in some *A. thaliana* ecotypes by introducing functional *A. lyrata SCR* and *SRK* genes, while several other ecotypes have remained self-fertile [31,33,35,40,43]. Notably, transgenic *A. thaliana* expressing *A. lyrata SCRb-SRKb* in the C24 ecotype were self-incompatible and produced very few seeds, while the same combination in the Col-0 ecotype resulted in full seed production with selfing [33,35,40]. On the other hand, the addition of *ARC1* with *A. lyrata SCRb-SRKb* in the Col-0 ecotype resulted in self-incompatible plants with reduced seed set [43].

In this study, we tested the reconstitution of the *SCR-SRK-ARC1* signaling pathway in self-compatible *A. thaliana* using new sets of self-incompatibility genes from two other species, self-incompatible *B. napus* and *A. halleri*. Our results showed that self-pollen rejection was achieved only in the *A. thaliana AhSCR_13_-AhSRK_13_-AhARC1* Col-0 lines. The other combination of the *AhSCR_13_-AhSRK_13_* transgenes was not sufficient to restore self-incompatibility in *A. thaliana* Col-0 ecotypes, similar to that previously observed [43]. Interestingly, only flowers from the *AhSCR_13_-AhSRK_13_-AhARC1* strongly self-incompatible Col-0 plants displayed an approach herkogamous phenotype, which had been previously detected with the *SCRb-SRKb-ARC1* transgene combination [43]. The self-compatible *A. thaliana* Col-0 *AhSCR_13_-AhSRK_13_-AhARC1* plants maintained the same floral phenotype as wild-type Col-0 plants, indicating that the approach herkogamous phenotype is associated with the intensity of self-incompatibility of the transgenic plants. The expression of *SRKb* was previously found to enhance pistil elongation and stigma exertion in the rdr*6* mutant background [52]. Thus, the self-incompatibility signaling transduction mediated by *AhSCR_13_-AhSRK_13_-AhARC1* might also activate downstream components that are regulating pistil development.

As nearly half of the *A. thaliana* Col-0 *AhSCR_13_-AhSRK_13_-AhARC1* plants still exhibited a self-compatible phenotype, quantitative RT-PCR was conducted to test if the expression levels of the transgenes were correlated with the ability of self-pollen rejection. However, there was no clear relationship between the relative expression levels of the three transgenes and the intensity of self-incompatibility response. Thus, the trigger for self-pollen rejection in the transgenic *A. thaliana* Col-0 *AhSCR_13_-AhSRK_13_-AhARC1* lines did not appear to be dependent on the expression levels of the transgenes. Unexpectedly, no self-incompatible lines were generated in both the *A. thaliana* Col-0 and C24 ecotypes with the transformation of *BnSCR_1_-BnSRK_1_* or *BnSCR_1_-BnSRK_1_-BnARC1* genes. These *B. napus* transgenes displayed similar expression patterns to the *A. halleri* transgenes, and so their relative expression levels did not appear to account for the lack of a self-incompatibility phenotype. A potential reason may be the design of these transgenes, where only coding sequences were cloned and driven by promoters from other genes, potentially impacting their expression. As the *BnARC1* transgene was previously shown to be functional in the *A. thaliana* Col-0 background [43], it is more likely that *BnSCR1* or *BnSRK1* are not functional in *A. thaliana*. Similar, the *BnSCR1* construct was found to be functional in *B. napus* [46], and so *BnSRK1* would be the only previously untested construct. One other possible reason is that *Arabidopsis* and *Brassica* species may not completely share the same SRK-mediated self-incompatibility signaling pathway. These differences may potentially be due to *B. napus* SRK_1_ activity, as *B. napus* ARC1 was previously shown to interact with *A. lyrata* SRK, and the *A. lyrata SCRb-SRKb* and *B. napus ARC1* transgenes produced self-incompatible Col-0 transgenic plants [43,53]. Ideally, the potential activity of the *B. napus* transgenes could be tested by reciprocal pollinations between the *A. thaliana* Col-0 *BnSCR1-BnSRK1-BnARC1* lines and the *B. napus* S1 haplotype transgenic line [46]. However, large physical differences in pollen and pistil sizes between these species (Supplementary Figure S2, [38]) as well as potential complications from interspecies crosses may obscure any self-incompatibility reactions.

There are several pieces of evidence that would support the idea of differences in the *Brassica* and *Arabidopsis* SRK-mediated signaling pathways. *A. thaliana* diverged from *A. lyrata* about 10 MYA and diverged from *Brassica* approximately 24 MYA [54,55,56,57,58]. As would be expected within the *Arabidopsis* genus, the inactivated *SCR* and *SRK* alleles in *A. thaliana* share high sequence similarity with the functional *S*-haplotype genes in *A. lyrata* and *A. halleri* [28,30,31,34,35,50,59,60]. In contrast, there is high variation in their sequences when compared to *Brassica SP11/SCR* and *SRK* alleles, and so this variation could lead to differences in SRK interactions with other downstream components (the ARC1 interaction is conserved). The ability of the *SCRb-SRKb* transgenes to restore self-incompatibility in the C24 ecotype (i.e., no ARC1) supports the idea that C24 plants might employ other unknown self-incompatibility signaling players; for example, a strong calcium flux mediated by glutamate receptor-like channels has been shown to occur in self-incompatible C24 stigmas [33,35,40]. In addition, while MLPK is required for self-incompatibility both in *B. rapa* and *B. napus* [17,18], an *A. thaliana* homolog of *MLPK* (*APK1b*) was found to be unfunctional in regulating the transient self-incompatibility response observed in *SCRb-SRKb* Col-0 lines [42]. Finally, there are differences in the cellular responses that take place in the stigmatic papillae of *Brassica* and *Arabidopsis* species, such as autophagy and the trafficking of vesicles and multivesicular bodies [16,61]. The potential redundancy shaped by genome triplication and the formation of distinct subgenomes in *Brassica* species may have increased robustness in the self-incompatibility system, allowing further evolution of the underlying signaling pathways and distinction from *Arabidopsis* species [62,63]. 

In conclusion, we have demonstrated that the previously untested *A. halleri* S13-haplotype genes, along with *AhARC1*, can restore the self-incompatibility phenotype in *A. thaliana* Col-0 plants, and that *AhARC1* is required along with *AhSCR_13_-AhSRK_13_* for self-pollen rejection in the Col-0 ecotype. The generation of these self-incompatible *A. thaliana AhSCR_13_-AhSRK_13_-AhARC1* Col-0 plants will be a very useful resource for further studies on sporophytic self-incompatibility in *A. thaliana.* Our results also show that the equivalent set of self-incompatibility genes from *Brassica* (*BnSRK_1_*, *BnSCR_1_* and *BnARC1*) was not sufficient to establish self-incompatibility in both the *A. thaliana* Col-0 and C24 ecotypes. These observations indicate that the transfer of self-incompatibility into *A. thaliana* is based on the phylogenetic relationships with the transgene donor, and it would be good to verify this by testing other combinations of *SP11/SCR* and *SRK* alleles from different *Brassicaceae* species. Since the *Brassica*-specific *MLPK* gene has been proven to be necessary for self-incompatibility in *Brassica* species [17,18], it would be worth transforming *B. napus SCR-SRK-MLPK-ARC1* all together into *A. thaliana* to determine if *MLPK* is the missing component for reconstructing the *Brassica* self-incompatibility pathway. As well, the reciprocal experiment of transferring *Arabidopsis SCR* and *SRK* genes into self-compatible *B. napus* would be quite interesting in order to see if the *Arabidopsis SCR* and *SRK* genes can restore self-incompatibility in a *Brassica* species, or if the diversification of the self-incompatibility pathway extends in both directions.

## 4. Materials and Methods

### 4.1. Plant Materials

The wild-type *A. thaliana* Col-0 and C24 plants and the transgenic lines were grown under long-day conditions in a greenhouse with a 16-h light/8-h dark photoperiod at 22 °C. The transgenic T1 plants were selected from seeds collected following floral dipping by being plated on 1/2 MS medium containing kanamycin.

### 4.2. Vector Construction and Plant Transformation

The functional promoter and CDS of *BrSP11-47* (the CDS sequence is the same as *BnSCR_1_*) was amplified from the pCAMBIA2301-1+4 vector [46]. And the Nopaline synthase polyadenylation signal (NosT) was amplified from the pCAMBIA2301 vector used as the terminator of *BnSCR_1_* [64]. These two fragments were subcloned into the *Bsp120I* and *Psp1406I* sites of the pORE_O4 binary vector, respectively [65]. The SLR1 promoter is expressed in particular in stigma of *Arabidopsis*, and was amplified from *B. napus* ‘Westar’ genome DNA using forward and reverse primers with *KpnI* and *XbaI* sites [66,67]. The full-length CDS of *BnSRK_1_* was cloned from ‘Westar’ cDNA with primers carrying *XbaI* and *BstEII* sites. The SLR1 promoter and *BnSRK_1_* CDS were joined using the *XbaI* site by being cloned into the pCAMBIA2301 vector, then the SLR1pro-*BnSRK_1_* fragment was amplified from the medium vector with primers with *Cfr9I* and *NotI* sites for directional cloning into the pORE_O4 vector. A 989 bp-terminator of *BnSRK_1_* was obtained from ‘Westar’ genome DNA and was cloned into the pORE_O4 vector at the *NotI* site. For the reconstitution of the *BnSCR_1_-BnSRK_1_-BnARC1* construct, the full-length CDS of *BnARC1* was amplified from ‘Westar’ cDNA with primers with *SalI* and *KpnI* restriction sites. *BnARC1* was also driven by the SLR1 promoter, which was amplified using primers with *HindIII* and *SalI* sites that were then cloned into the pCAMBIA2301 vector to be joined. The SLR1pro-*BnARC1* fragment was further cloned by primers carrying the *KpnI* site and ligated into the pORE_O4 vector.

All promoters and genes from the *A. halleri S_13_* haplotype [47] and *AhARC1* together with its promoter were synthesized by Invitrogen GeneArt (Thermo Fisher Scientific, Waltham, MA, USA). It should be noted that *AhSRK_13_* was driven by *AhSRK_20_* promoter, since *AhSRK_13_* and *AhSRK_20_* were codominant in *A. halleri* [41]. *AhSRK_20_* promoter, *AhARC1*pro-*AhARC1*, *AhSCR_13_*pro-*AhSCR_13_* and *AhSRK_13_* were ligated into the pORE_O4 vector using the *Cfr9I-NotI*, *XbaI*, *Bsp120I* and *NotI-Acc65I* sites or pairs of sites in the order they were mentioned (see Supplementary File S1 for further details and sequences). *AhARC1* was not cloned into the pORE_O4 vector for the combination of *AhSCR_13_-AhSRK_13_* transgenes. All PCR products were amplified with PrimeSTAR HS DNA polymerase (Takara, Kusatsu, Shiga, Japan), and the PCR products were then ligated to pGEM-T Easy (Promega, Madison, WI, USA) and sequenced. These constructs were introduced into *Agrobacterium tumefaciens* GV3101 host cells. PCR primers used in vector construction steps are listed in Supplementary Table S1. The plant transformation was performed following the floral-dip method [49,68].

### 4.3. Pollination Assays

All the floral buds of the *A. thaliana* Col-0 and C24 transgenic plants were emasculated and covered with a paper bag one day before anthesis to avoid pollen contamination. Pollinations were performed the next day (day of anthesis). The pistil was cut at the peduncle 4 h after pollination, then fixed for 2 h in ethanol–acetic acid (3:1), softened in 1 N NaOH at 60 °C for 1 h and stained with 0.01% (w/v) decolorized aniline blue for 2.5 h in 2% (w/v) K_3_PO_4_. Pistils were gently squashed onto a microscopic slide glass by placing the cover glass over the pistils. Samples were examined under a fluorescence microscope (Eclipse 80i, Nikon, Minato, Tokyo, Japan).

### 4.4. Quantitative RT-PCR Assays 

Total RNAs were extracted using the SV Total RNA Isolation System (Promega Madison, WI, USA). The RNA samples were quantified using a NanoDrop Spectrophotometer (Nanodrop Technologies, Wilmington, DE, USA), and 1 µg RNA of each sample was used to synthesize the first-strand cDNA with a Thermo RT kit (Thermo Fisher, Waltham, MA, USA). The cDNA samples were used as templates of the qRT-PCR assays. The qRT-PCRs on the transgenic lines were performed using cDNA of the mature buds and 2× Power SYBR green (Toyobo, Osaka, Osaka Prefecture, Japan). qRT-PCRs were then conducted on the CFX96 Touch Real-Time PCR Detection System (Bio-Rad, Hercules, CA, USA), and the conditions used were a pre-denaturation at 95 °C for 3 min, followed by a three-step cycle of 10 s denaturation at 95 °C, 10 s annealing at 65 °C and a 30 s extension at 72 °C for 47 cycles with a melt curve. The results were calculated with CFX Manager Software (Bio-Rad Hercules, CA, USA). Primers used in qRT-PCR analysis are shown in Supplementary Table S2.

### 4.5. Accession Numbers

*BnSCR_1_* gene (Genbank accession AB270773), *BnSRK_1_* gene (Genbank accession AB270771), *BnARC1* gene (Genbank accession AF024625). Sequence data and accession numbers of *B. napus* can be found in http://www.genoscope.cns.fr/brassicanapus/. Sequence and accession numbers information for *A. thaliana* and *A. halleri* genes used in this study were obtained from TAIR and Phytozome.

## Figures and Tables

**Table 1:** Phenotypes of transgenic *Arabidopsis thaliana* Col-0 plants.

	Self-Incompatible			Total No. T_1_ Plants Tested
Transgenes	Strongly	Moderately	Self-Compatible	
*AhSCR_13_-AhSRK_13_*	0	0	30	30
*AhSCR_13_-AhSRK_13_-AhARC1*	4	16	14	34
*BnSCR_1_-BnSRK_1_*	0	0	21	21
*BnSCR_1_-BnSRK_1_-BnARC1*	0	0	12	12

**Table 2:** Phenotypes of transgenic *A. thaliana* C24 plants.

Transgenes	Self-Incompatible	Self-Compatible	Total No. T_1_ Plants Tested
*BnSCR_1_-BnSRK_1_*	0	13	13
*BnSCR_1_-BnSRK_1_-BnARC1*	0	9	9

## Supplementary Materials

The following are available online at https://www.mdpi.com/2223-7747/8/12/570/s1. **Figure S1.** Pollen tube germination and growth on stigmas of wild-type *A. thaliana* Col-0 and transgenic Col-0 plants following reciprocal crossings. (**A,B**) Wild-type *A. thaliana* Col-0 stigma pollinated with pollen from transgenic strong self-incompatible Col-0 *AhSCR_13_-AhSRK_13_-AhARC1* line 2. (**C,D**) Transgenic strong self-incompatible Col-0 *AhSCR_13_-AhSRK_13_-AhARC1* line 2 stigma pollinated with pollen from wild-type *A. thaliana* Col-0 plant. Differential interference contrast (DIC) and aniline blue-stained images are shown for each sample. SC: self-compatible; SI: self-incompatible. Bars = 100 μm. **Figure S2.** Flowers from wild-type *B. napus* ‘Westar’, *A. thaliana* Col-0 and C24, and transgenic *A. thaliana* Col-0 and C24 plants. (**A,B,H**) Flowers of wild-type *B. napus* ‘Westar’, *A. thaliana* Col-0 and C24 plants. (**C–G**) Flowers of transgenic *A. thaliana* Col-0 lines: *BnSCR_1_-BnSRK_1_* line 12, *BnSCR_1_-BnSRK_1_-BnARC1* line 9, *AhSCR_13_-AhSRK_13_* line 5, and *AhSCR_13_-AhSRK_13_-AhARC1* lines 2 and 21. (**I,J**) flowers of transgenic *A. thaliana* C24 lines: *BnSCR_1_-BnSRK_1_* line 90, and *BnSCR_1_-BnSRK_1_-BnARC1* line 16. SC: self-compatible; SI: self-incompatible. Bar = 1 mm in (**A**) and bars = 200 μm in (**B–J**). **Table S1.** Primers used in vector construction. **Table S2.** Primers used in qRT-PCR assay. **Supplementary File S1.** Map and sequences for *AhSCR13-AhSRK13-AhARC1*.
