# Supplementary material for: Generation of Transgenic Self-Incompatible Arabidopsis thaliana Shows a Genus-Specific Preference for Self-Incompatibility Genes
Source: Plants (Basel). 2019 Dec 4;8(12):570. doi: 10.3390/plants8120570 (PMC6963867; doi:10.3390/plants8120570)
Supplement: Supplementary file 1 [file plants-08-00570-s001.pdf]

plants MDPI

Supplementary File S1. Map and sequences for AhSCR13-AhSRK13-AhARC1.

pORE_O4 vector: Coutu et al. pORE: a modular binary vector series suited for both monocot and dicot plant transformation. Transgenic Research 2007, 16: 771-781.
A. halleri ARC1 sequence: retrieved from Phytozome.
A. halleri S-haplotype sequences: retrieved from GenBank (Goubet et al. Contrasted patterns of molecular evolution in dominant and recessive self-incompatibility haplotypes in *Arabidopsis*. PLoS Genet. 2012, 8(3):e1002495).

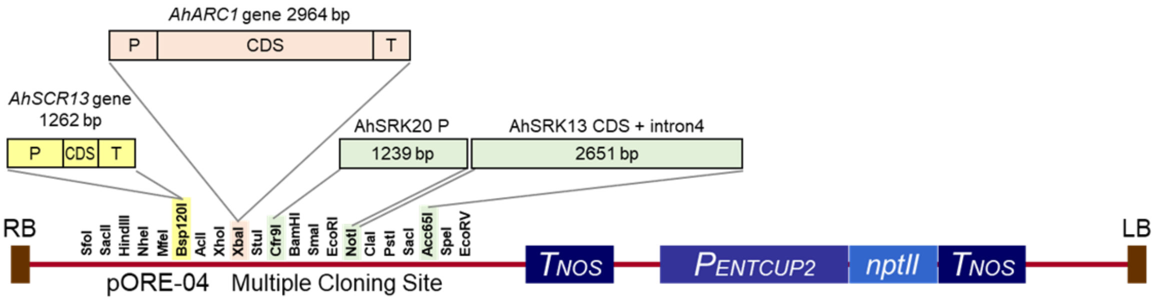

Arabidopsis halleri ARC1 gene sequences retrieved from Phytozome: Araha.20846s0006

>*AhARC1* Promoter-CDS-terminator *XbaI-XbaI*

TCTAGAcaattttacagttttgaattcaaagcaatttatttatcgtagcatactttttttttggataaatcctatcggctgatccggtcggtcagcctc
ggtaggaatgcaccgaagtgctacagagtggacttctcctgcggttcaacgattcaacgtagcatactttgcatcagtgggtttcaaaattgcaata
tggttttgacaccaataaacatcaacggtacataattgatctcaactaccagctaatacagagaatggtaagtgtgactaattatgatccgttacaat
gttacatacttttacttaaatgtcaacattcataacaacgaagaattttttttcagattttgccaaacgcgttttacaaatctcttctttgttctttcgcaccc
gcaaataaatttgttgtcgttgttcttacacagtccaagtcttaggcttctttgatttgacaagccacactggttttggagccatccatcgtcaacATG
GCCACCGAGGCGATATTCGCATCCTTACGACGGAGGAGCTCGCCGTCACTGGACGCATTTT
TGACAACAACCGTTGATCTCTCCGGCGTCCCTCTCATTCAAACCCTAGCTTCAATTTCAGCA
GAAATCGTCTCGTGCTTCCGCGGCGTACGTTTCTCCTTCCAACGCAGAAACTCACGTTCTCT
GATTCGGAAGATTGAGGTCTTGCTCGTCTTGTTTAAATACATCTCCGATGATTCAGGTTTGG
ATTCGACGGCAGTACTTTGCTTTAAGGAGCTCTATCTCTTCCTCCACCGTTCCAAATTCCTTC
TCCATTACTGTGCTCAGTCCAGTAAACTATGGCTCTTATTACAAAACCCATCGCTTTCAGAT
TTTTTCCATGTTTTGAATCGTAATATTTGTACCCTTTTGGATGTTTTACCCATCAACAGTCTTA
ATCTAAGCGACGACATTAGAGAACAAATCGAGCTTTTGCATCAACAATCCAGTAAATCGAC
GACACTGTTCGTTGATCACAACGACGACTTTCTACGGGACCAATTCTATTCGTTTCTCCACG
GATTTGAGAAGGGTCAGATACCCAATTCGGAAGAATTGAGATCTTTCTTCGTAGAGAAATT
GGAGATTAGGGATCCGAAAAGTTGCAGTGATGAAATCGAGTTTCTTGAAGAGCAGATAGT
GAATCATGATTGTGATGATTTAGAGCCTACAAGGTCGTTGATTAACGGGTTTGTGGCTATCA
CACGTTATTGCATGTTTTTGTTGTTTGGGTTTGAAGATGATGGAGAGTGGAGTATCGAGAAC
TTGAAGAAACAGAGGAAATGTTTTATTGCGGAGGAGATTGTGGACACGTTTATGACGCTAC
CAAAGGATTTTGTTTGCTCTATCTCTCTTAGTTTGATGAAGGATCCTGTGATTGTTTCCACTG
GACAAACTTATGATCGGAGCTCCATAGTTAGGTGGTTTGAAGAAGGTCACTCTACTTGTCCC
AAAACAGGGCAAAAGCTTGTGGACTCTTCGTGTATTGTTCCTAATCTAGCTTTGAGAAACTT
GATAACACGTTGGTGCGCAGCAATGGAGTTTGAGGATTCACCAAACGAGTCTCCTGCTTTA
GTTCTTCAAACAAGAGCTTCAATGGAAGCAACTAAAGCCACTGTGTCGATTCTCATACAAA

ATCTAGCTAGTGGGTCAGAATTAGCTCAGATAGTGGCGGCACGAGAAATTCGTCTTCTAGC
CAAAACAGTTAGGAAAAGAGGTCTATTGATCGCGGAAGCAGGTGCAGTCCCGCATTTGTGT
AGACTTCTTAAATCCAAAAACGCTGTTGCGCAAGAGCATTCCGTTACAGCGATGCTTAATTT
GTCTGTCTGCGAGGAAAACAGAAGCCTGATCATGGAGGAAAATGATTGCCTTGAGTCGATT
GTGAGCGTTGTCGCCTCGGGTCTTACTTTGGAAGCTCAAGGAAACGCAGCAGCCACATTGT
ATAGTCTTTCCACAGTACATGAATATAAGAAACGGATCGCAAATGTAGATGGCTGCATCAA
ATCACTTGCATCGCTGTCGAGGAATGGAAAGCCGAGAGGGAAAAAAGATGCACTCAACGC
TTTATATGGCATATGGTCACATCCGGATAATTGCAGCCAGATGATCAACTCGGGAGGAGTG
TCCGCTGTCGTGGGAGCTTTGGCGGATGAGGACGAGGCTGTGACGGAGAGAGCGGCAGTA
GTGTTGGGGGTAGTAGCGAATCATTCTTTAGGAGCAGAGACTATAGGGAGAGAGGAATCA
GCTGTGGCTGGACTCATTGAATTGATGAGATGTGGAACACCAAGAGGCAAAGAAAATGCG
GTTGCTACTTTGTTACATCTCTGCATAAACGGTGGAACGGTTGTGGTGGAGAAGGTGGTGAG
AGCACCGGCTCTTTCTGATTTAACCCAAAAGCTTTTGCTAACGGGTACAAATCGAGCTAAG
CGGAAAGCGAGTTCATTCCTCGCTTTGGTTCGTAAGGGGTGCGAAAACACAGCGATGATGC
GGTCTGGTAATAGAGAAGGGAGTTTTAGAACCCATGTCTCCCTACCCATCTCTATACCTGTA
TCTGTGTTGTGAtcttacatacatacatacgcacataagccttagagaataatgttaatggggaagatataagagttttgttaagcgatgtt
tatgtattgcctagaaatattatgtgatgtaataagctataagtttattgatacaatcggttgtatagcttgtttgtctgaatggaaacgcaagtgaca
attcggtatcgattcagtcatcttcactctggtaaaaaacaaattgtatctaagacttttttttaggtcgcatcaatttgtacaatggtttgcatcaagtt
aggtaaaaaaagtgtatctaagactttgaatattaatctaagtgttttccatatgttttacattacattacattagatcgTCTAGA

Arabidopsis halleri SCR13 gene sequences retrieved from GenBank: FO203478

>*AhSCR13* Promoter-CDS-terminator *Bsp120I-Bsp120I*

GGGCCCtttccgccagcaccaaggagacatattgagtattgagggccgaattttattcttataaagaaagaccgcaagctagtgcttcatcag
cttcctccaagggccgtgtctgcctcagtgcctccagtactgttttataaaaatttacacctcgagcagtatgatatactgatatgtttcatttgcttgta
gacgtagccaacacatggttggtaaaccacgtaaacttgttattttgtgtctctattagtttttactttttccgtcataacatatatattgacatgttcaact
tgtttaaataactataattagtacaaaaagaaacatgaaaaagtgatttttctgataagaactatccaaaaataaaggattgatccatcaatcttgtt
gacttggcctcacccttatatttccccctaatttaattgaaacaatttatttactgtcttggatcccctatatataacgaaatcaagacaaacaatagattt
gtgaaagtaaaaaaagacagtctttaatcacttttcttgaatcATGAGACGTGCTACATTGTTCATAGTTTCTTTCGTT
TTCATATCCCTTTTATTGAGTAATATTCAAGgtaatatattaacactttttgagttctgattggttgttcgatcgggatattt
taaaaactaattttaactaagaatataactagtgtttctgattgatatgaaatgatagATGGGGAAGCTCTGACGAAAAAGAA
GTGCTTCGAGGCGAAAGTATTCCCCAGCGGAAAATGTGGCAGAGACGGAAACTACAACTG
CAAAAAAGAGTATAAGAATAGTCGCGTGAAACCTACTAATTGCAAGTGCTTCAGTAATTTT
GATGAAACACGTTTGTGCAATTGTACATTTTGCTAAgttttactatataactatcacaaaaattctttattaataatgaa
gggagaagcaataaaatttagctgcaataagagagaaactgtgagattgtaaggtgttgccatgccggacaatatagtcatattagtgtagtgct
atttcagatttaactactttgttttcgtgaaatcaataaaattagatgttataaccacattaacttgattagtatcttagtcacaactttttgtgctcaact
atcgctcactaatgatatagtatctcttaaacactgtattcctggaaatattagaaggcgtaaaataatcaatttgataaccgttaaagggcttgtcga
taaaccggacagctcGGGCCC

Arabidopsis halleri SRK20 promoter sequences retrieved from GenBank: KJ772396

>*AhSRK20* Promoter + 5’UTR *Cfr9I-NotI*

CCCGGGttaacgacggatttttttcgttgtgaatactgtgccaaatatctcacgtacaacacatgatcatccctcgatgattcgttgttaataaact
agagacatatctagtcatggaaacactaacgatgaatgtttgtagtagaaaaaggtgattttatacatgtatatgtattctacgtttatagcaaacat

tcaccaaattttgacaatgtctactccgtcgttaaaacgattgcaaaatcgtcataaatatattgctattttgtagttatataacgaagttaaagttgaa
ggttgtgtaaattggtttttaaaaagtctacttaaaaaacactatttgcttccaaatgttttcgttgaattcaaaatatctataaatctaggcaatggatg
tattgatgaactgaaaaaaaaaaagtattgatgaactgatgcaaaaacgcccaaagtgatagagaaatacataagtggtccacaaaatttcattc
acgaggacttgaggactatgttcaactactgaatgccatttgtcagtttctgccggctttggtttttgattagagtttggtttaagtgtcaactttaaaat
ttcagaaaataaaaatgttttaaccatataaatctataattcatttttcttagttaggtttggttttgtttacctgttttcatataattcgtttaacagattga
ttatttttgaaaatttagttttaatacgattttactgaattcatttaagtcaaaccaattaagttggactctctactatgtacgattctgaatcttataaaatt
gtcgaccaaaaggtacgacaatgttctaacatactttgaaatttgaaagtatggaaggatgcccttgagaacaccacatttgcaaacatatgttatg
ttggcagcgtgggaaaataatttaacgtgtagatttaaaggttgtttttttttttttgataagagatttaaaggttgttgttgaccaatgataaaagttt
gtggaaatgaatttattgaagggacgaaaaagtcatggaatatggaaacaacattttgcttgtctgttaggtccgtgataataatgtagtttcagat
tttcttgccaacttaggattaagatataaatatatgcaatttaaaatcgaaaaccactttcactcgtttaggtcaatcgtacgtgatatttttcataaatat
aaaaagaaaactgatcgaagaagaagaaagaaaGCGGCCGC

Arabidopsis halleri SRK$_{13}$ gene sequences retrieved from GenBank: FO203478

>*AhSRK*$_{13}$ CDS + intron4 NotI-Acc65I
GCGGCCGCgagagATGAGAGCTCTACCAAACAACCACCATTTTTACATCCTCGTTATCTTTTTT
CTGCTTCGTTCTGCATTACCCATCAATGTCAATACTTTGTCGTCAACAGAATCCCTGACAAT
CTCAAGCAACCGAACCATTGTGTCTCTTGGTGATGTCTTCGAGCTTGGTTTCTTCAACCCCAC
ACCAAGCTCTCGAGATGGTGATCGTTGGTATCTCGGGATTTGGTACAAGGAAATCCCTAAG
AGAACCTATGTATGGGTTGCCAACAGAGACAATCCGCTTTCAAATTCCACTGGAACCCTCA
AAATCTCTGATAATAATCTCGTCCTCGTCGATCAATTCAACACCCTTGTTTGGTCGACGAAT
GTGACTGGAGCTGTGAGATCTCTAGTGGTGGCAGAGCTTCTCGCTAACGGAAATTTGGTGCT
TAGAGACTCCAAAATCAACGAGACAGATGGATTCTTGTGGCAGAGTTTCGATTTCCCAACA
GATACTTTACTTCCGGAGATGAAACTTGGTTGGGATCTAAAAACAGGAGTCAATAAATTCC
TTAGATCCTGGAAAAGCCCATATGATCCCTCAAGTGGGGATTTCTCATACAAACTTGAAAC
TCGAGAGTTCCCAGAGTTTTTCCTATCGTGGAGCAACTCGCCAGTGTACCGTAGCGGTCCGT
GGGAGGGATTCCGGTTTAGTGGCATGCCAGAAATGCAACAATGGACAAACATCATTTCCA
ATTTCACGGAGAACAGAGAGGAGATCGCTTACACTTTCCGAGACACTGACCAAAACATCT
ACTCAAGATTAACAATGAGTTCCTCAGGGTATCTACAACGATTTAAGTGGATTTCGAATGG
AGAAGATTGGAACCAACATTGGTACGCACCAAAAGACAGATGTGATATGTATAAGAAGTG
TGGGCCCTACGGTATTTGTGACACGAACAGCTCACCGGAGTGTAACTGTATTAAAGGGTTT
CAACCGAGGAATCTGCAGGAGTGGTCGTTGAGAGATGGATCGAAAGGGTGTGTGAGGAAG
ACGCGACTGAGCTGCAGCGAGGATGCGTTTTTCTGGCTGAAAAATATGAAGTTGCCAGATA
CTACGACAGCGATCGTGGACAGGAGACTTGGTGTAAAAGAATGTAGAGAGAAGTGTCTTA
ATGATTGTAATTGTACAGCCTTTGCAAATGCGGATATCCGTGGTTCAGGTTGTGTGATTTGG
ACGGGAGATCTCGTGGACATACGGAGCTACCCCAATGGCGGTCAAGATCTTTGCGTCAGAC
TGGCTGCTGCTGAACTCGAGGAGAGAAACATAAGAGGAAAAATCATAGGTTTATGTGTTGG
AATCAGCCTCATCCTTTTTCTGAGTTTCTGCATGATCTGTTTTTGGAAAAGGAAACAGAAGC
GACTAATAGCCTTAGCAGCACCTATCGTGTACCATGAGAGAAATGCAGAGTTGTTAATGAA
CGGGATGGTAATATCAAGCCGGAGACGCTTGTCTGGAGAGAACATAACAGAGGATTTGGA
ACTTCCACTCGTTGAACTGGACGCTGTTGTCATGGCCACTGAAAATTTCTCCAATGCTAACA
AGGTTGGACAAGGTGGTTTTGGTATTGTTTACAAGGGAAGATTACTTGACGGGCAAGAGAT
CGCAGTGAAAAGGCTGTCTAAGACTTCACTTCAAGGGACCAATGAGTTCAAAAACGAAGT

GAGGCTAATTGCGAAGCTTCAACACATAAACCTTGTCCGACTTCTTGGCTGTTGTGTTGAGG
TGGACGAGAAGATGCTGATCTATGAGTATTTGGAGAATCTAAGTCTTGATTCTTATATTTTT
GgttagtacctcaccttttaagaagctatgttctacaattaattgtctttagaaataatgtactaatctgtttgcgattggtttgtagACAAAAAT
AGAAGCTGGAAGTTAAATTGGCAAATGAGATTTAATATTACTAATGGTATTGCTCGAGGAC
TTTTATATCTTCACCAAGATTCACGATGTAGGATTATCCACAGAGACTTGAAAGCAAGCAA
TGTCTTGCTTGATAAAGATATGACTCCGAAAATATCAGATTTCGGGATGGCTAGGATCTTTG
GACGGGAGGAGACAGAAGCAAACACGAAGAAGGTGGTCGGAACTTATGGCTATATGTCTC
CAGAATATGCAATGGATGGAGTTTTCTCGATGAAGTCGGATGTTTTCAGCTTTGGGGTCTTG
CTTCTCGAGATTATAAGTGGAAAGAGGAACAAAGGATTCTACAACTCTGACAATGACCTTA
ATCTTCTCGGTTGCGTGTGGAGGAATTGGACAGAAGGAAAAGGTCTAGAGATCGTAGATCC
GATAATCCTAGAGTCCTCATCATCAACGGTCATACTACAAGAAATATTAAAATGCATGCAA
ATCGGTCTCTTGTGTGTTCAAGAACGTGCAGAAGACAGACCAAGAATGTCTTCGGTGGTGG
CAATGCTCGGAAGCGAAACGGCAGTGGTTCCTCAGCCTAAACTACCTGGTTATTGCGTTGG
GAGAAGTCCTCTTGAGACTGATTCATCGCGGAGTAAACAGCATGACGACGAATCGTGGACT
GTGAACGAAATCACCCTATCGGTCATCGACGCTCGATAAGGTACCC

Supplementary Figures

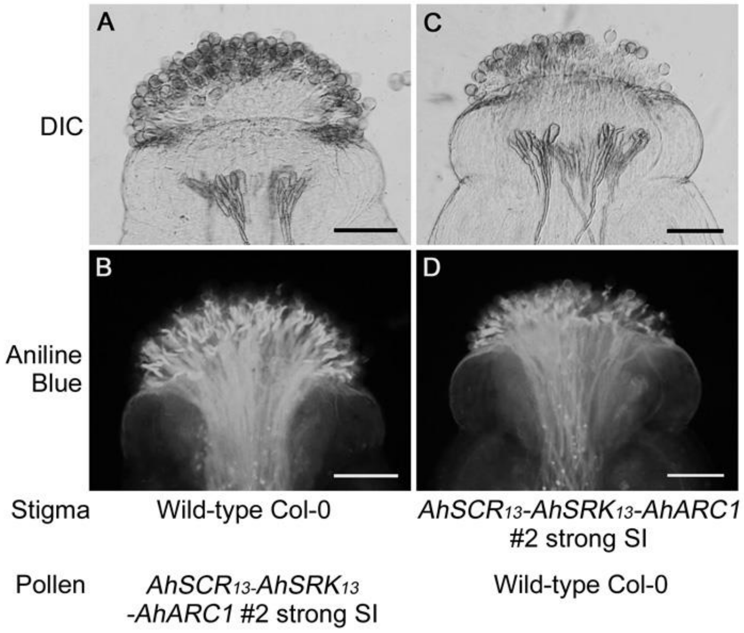

Figure S1. Pollen tube germination and growth on stigmas of wild-type *A. thaliana* Col-0 and transgenic Col-0 plants following reciprocal crossings. (**A**) and (**B**) wild-type *A. thaliana* Col-0 stigma pollinated with pollen from transgenic strong self-incompatible Col-0 *AhSCR*$_{13}$-*AhSRK*$_{13}$-*AhARC1* line #2. (**C**) and (**D**) transgenic strong self-incompatible Col-0 *AhSCR*$_{13}$-*AhSRK*$_{13}$-*AhARC1* line #2 stigma pollinated with pollen from wild-type *A. thaliana* Col-0 plant. Differential interference contrast (DIC) and aniline blue-stained images are shown for each sample. SC: self-compatible; SI: self-incompatible. Bars = 100 μm.

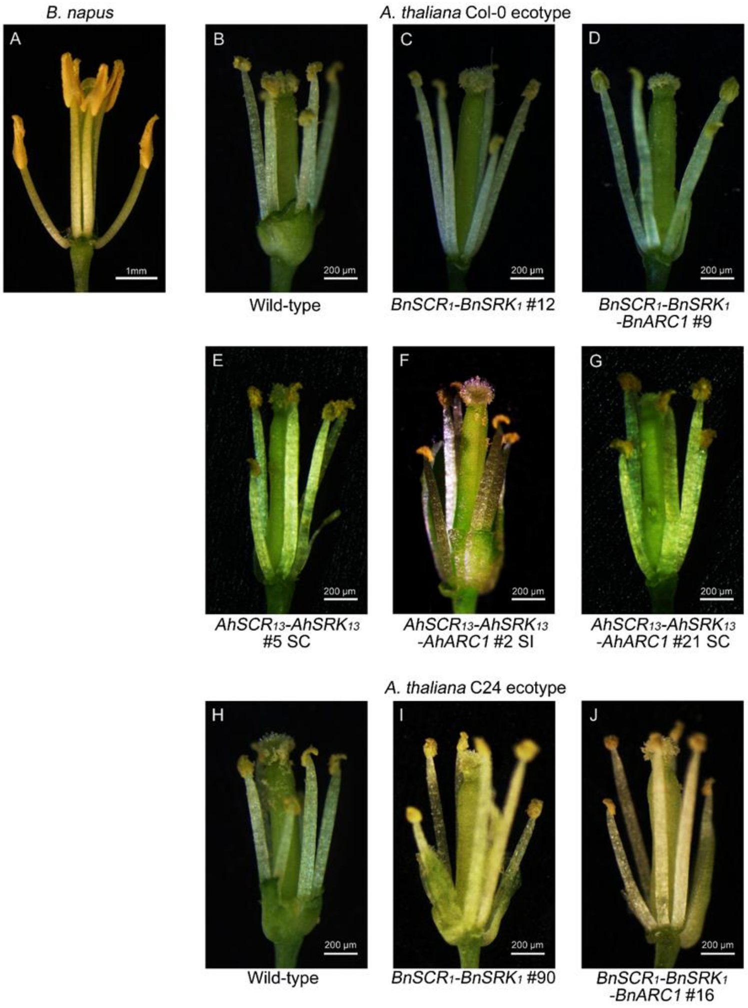

Figure S2. Flowers from wild-type *B. napus* 'Westar', *A. thaliana* Col-0 and C24, and transgenic *A. thaliana* Col-0 and C24 plants. (**A**), (**B**) and (**H**) flowers of wild-type *B. napus* 'Westar', *A. thaliana* Col-0 and C24 plants. (**C**) to (**G**) flowers of transgenic *A. thaliana* Col-0 lines: *BnSCR*$_1$-*BnSRK*$_1$ #12, *BnSCR*$_1$-*BnSRK*$_1$-*BnARC1* #9, *AhSCR*$_{13}$-*AhSRK*$_{13}$ #5, and *AhSCR*$_{13}$-*AhSRK*$_{13}$-*AhARC1* #2 and 21. (**I**) and (**J**) flowers of transgenic *A. thaliana* C24 lines: *BnSCR*$_1$-*BnSRK*$_1$ #90, and *BnSCR*$_1$-*BnSRK*$_1$-*BnARC1* #16. SC: self-compatible; SI: self-incompatible. Bar =1 mm in (**A**) and Bars = 200 µm in (**B**) to (**J**).

Supplementary Tables

Table S1. Primers used in vector construction.

Primer name	Sequence	Use	PCR product length
S1E1-Bsp120I	GGGCCCTACGACCTGCTGATATTCTCC	*BrS47-P* + *BnSCR1 CDS*	2160bp
S1E4-Bsp120I	GGGCCCCTAACACAATTTACATACACAAGAATAA	*BrS47-P* + *BnSCR1 CDS*	
NOS-1-Psp1406I	AACGTTGTTTCTTAAGATTGAATCCTGTTG	*2301-NOS*	231bp
NOS-2-Psp1406I	AACGTTCCCGATCTAA CATAGATGACA	*2301-NOS*	
SLR-PR1-KpnI	GGTACCGGATCCCTGGGTCATTGCT	*SLR1-P*	1511bp
SLR-PR2-XbaI	TCTAGACTCTCTCTTCACCACTTTAATTTTC	*SLR1-P*	
SRK$_1$-CDS-1-XbaI	TCTAGAATGAAAGGTGTACGAAACATCTATG	*BnSRK*$_1$ CDS	2556bp
SRK$_1$-CDS-2-BstEII	GGTTACCTTACCG GGCATCGATTACTGAGC	*BnSRK*$_1$ CDS	
SLR-PR1-Cfr9I	CCCGGGGGATCCCTGGGTCATTGCT	*SLR1P+BnSRK1 CDS*	4067bp
SRK$_1$-CDS-2-NotI	GCGGCCGCTTACCG GGCATCGATTACTGAGC	*SLR1P+BnSRK1 CDS*	

SRK$_1$-TL-NotI	GCGGCCGCTATGAAAGCTGTGAGAATGTTCATTT	*BnSRK$_1$* Terminator	988bp
SRK$_1$-TR-NotI	GCGGCCGCTAATAATGCTAAGGACTTAATTTGGGT	*BnSRK$_1$* Terminator	
ARC1-CDS-L-SalI	GTCGACATGGCCACTGATTCAGCAATG	*BnARC1 CDS*	1986bp
ARC1-CDS-R-KpnI	GGTACCTTATCTCTGTGTGTTCTGGTCGC	*BnARC1 CDS*	
SLR-PR1-HindIII	AACGTTGGATCCCTGGGTCATTGCT	*SLR1-P*	1511bp
SLR-PR2-SalI	GTCGACCTCTCTCTTCACCACTTTAATTTTC	*SLR1-P*	
SLR-PR1-KpnI	GGTACCGGATCCCTGGGTCATTGCT	*SLR1-P+BnARC1 CDS*	3497bp
ARC1-CDS-R-KpnI	GGTACCTTATCTCTGTGTGTTCTGGTCGC	*SLR1-P+BnARC1 CDS*	

Table S2. Primers used in qRT-PCR assay.

Primer name	Sequence	Use
BnARC1-qF	AAAGCCCCTCTCTTTCAAGC	qPCR
BnARC1-qR	TCAACGTAGTGCTGCATGTG	qPCR
BnSCR1-qL	ATGAAATCTGCTGTTTATAATGCTT	qPCR
BnSCR1-qR	TGTCCATACCCTTCGAATATAAC	qPCR
BnSRK-qL	CTCGTCTTCGTTGTCATGATTCT	qPCR
BnSRK-qR	GTTCATGTTGGATATTTTGAGGG	qPCR
AhSRK$_{13}$-qF:	TCGTTCTGCATTACCCATCA	qPCR
AhSRK$_{13}$-qR:	CAAATCCCGAGATACCAACG	qPCR
AhSCR$_{13}$-qF:	CAAGATGGGGAAGCTCTGAC	qPCR
AhSCR$_{13}$-qR:	TGCACAAACGTGTTTCATC	qPCR
AhARC1-qF:	CGTTCCAAATTCCTTCTCCA	qPCR
AhARC1-qR:	TGATGCAAAAGCTCGATTTG	qPCR
Actin2-F	GAAATCACAGCACTTGCACC	qPCR
Actin2-R	AAGCCTTTGATCTTGAGAGC	qPCR
TUB4-F	AACGCTGACGAGTGTATGGTT	qPCR
TUB4-R	CCAAAGGTAGGATTAGCGAGC	qPCR
